# A Case of Painful Growing Abdominal Wall Mass during Pregnancy Requiring Resection in the Second Trimester

**DOI:** 10.1155/2024/5881260

**Published:** 2024-01-08

**Authors:** Shlomo M. Stemmer, Cintia Gomes, Elyce H. Cardonick

**Affiliations:** ^1^Department of Obstetrics and Gynecology, Cooper Hospital, Camden, NJ, USA; ^2^Federal University of Santa Maria, Brazil

## Abstract

Desmoid fibromatosis (DF) is a rare and locally aggressive neoplasm. We present a case of a 28-year-old previously healthy multigravida who noticed a lump in her abdomen near the umbilicus two months before becoming pregnant. It underwent rapid growth during pregnancy, causing pain and discomfort. Targeted ultrasound of the area showed an irregular mass measuring 0.9 × 1.7 × 1.4 cm. The origin of the mass was unclear, suggesting a connection with the intra-abdominal contents. An MRI done three weeks later revealed a subcutaneous ovoid mass measuring 3.0 × 2.3 × 3.0 cm, which was significantly larger. Due to pain and rapid growth, surgical resection was done at 25 weeks of pregnancy. Histopathological examination revealed a desmoid tumor. The patient had an uneventful recovery and term vaginal delivery without complications. Hence, our case serves as evidence that DF tumors can be surgically managed during pregnancy with minimal to no complications.

## 1. Introduction

Desmoid fibromatosis (DF) is a rare soft tissue neoplasm characterized by the proliferation of monoclonal fibroblasts. While histologically benign, DF tumors can exhibit unpredictable behavior, as they invade surrounding tissues. Commonly found in various anatomical sites, such as the limbs, abdominal wall, and mesentery, these tumors can lead to functional impairment, pain, and potential life-threatening complications if they compress vital structures.

The incidence of DF is relatively low, ranging from 2 to 5 cases per million per year. It is more frequently observed in females, with a peak incidence during reproductive years [1]. The impact of pregnancy on desmoid tumors has been a topic of interest, but due to the rarity of the condition, the literature on its course, effects on future pregnancies, and management guidelines remains limited.

This case report presents a unique instance of a multigravida with an abdominal lump that exhibited rapid growth during pregnancy, necessitating surgical resection. The case is noteworthy for its presentation during pregnancy, the exceptionally fast growth rate of the tumor, and the requirement for surgical intervention due to pain. We also review the existing literature on this topic to shed light on the management and outcomes of DF tumors during pregnancy. By sharing this case and reviewing the literature, we aim to contribute to the understanding of DF in pregnancy and offer insights into its appropriate management.

## 2. Case Description

This is the case of a 28-year-old female, gravida 2, para 1, who presented to our office at seven-week gestation for routine antenatal care. She previously had one uncomplicated pregnancy and vaginal delivery. Her medical history was unremarkable, and she had no personal or family history of tumors.

**Figure 1:**
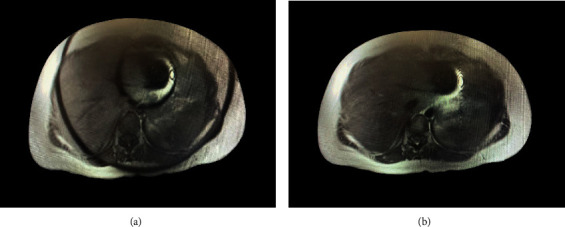
(a, b) MRI images of the mass.

**Figure 2:**
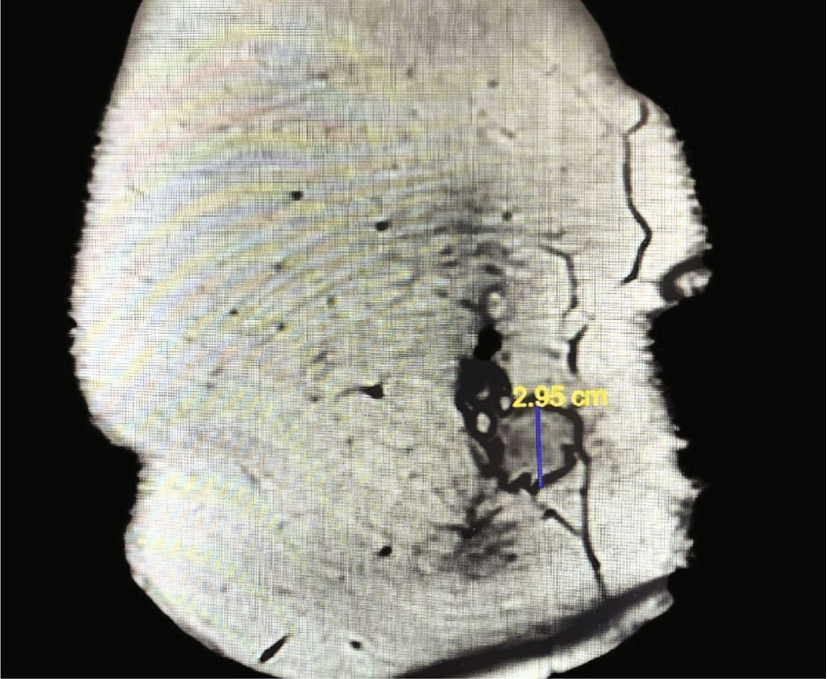
MRI measurement of the lesion.

The patient had noticed a lump in her abdomen near the umbilicus two months before becoming pregnant. During pregnancy, it showed rapid growth. Ultrasound and subsequent MRI were performed, revealing benign characteristics and no evidence of local or distant extension (Figures 1 and 2).

The uterus was palpable and appropriate for gestational age, and fetal heart tones were within the normal range. Maternal-fetal medicine and general surgeon specialists recommended expectant management during pregnancy and surgical excision after delivery. However, due to the continued growth and pain, the patient opted for surgical resection at 25 weeks of pregnancy. The mass measured approximately 3.5 cm and was resected under local anesthesia and IV sedation.

Histopathological evaluation of the resected mass revealed bland spindle cells in long fascicles with compressed blood vessels showing perivascular edema and focal extravasated red blood cells. Immunohistochemical stains were positive for beta-catenin, smooth muscle actin (SMA), and desmin. The diagnosis was consistent with desmoid-type fibromatosis, and the tumor had contact with the deep, medial, and focal superficial margins.

The patient had an uneventful recovery from the surgery and delivered vaginally at full term, giving birth to a healthy female infant with normal Apgar scores. Follow-up MRI seven months after the resection did not show any residual mass or recurrence. The management plan includes continuing with clinical and radiological surveillance.

## 3. Mass Characteristics

Prior to the resection, the mass measured approximately 3.5 cm and was located just below and lateral to the left of the umbilicus, subcutaneously. Physical examination revealed a small, round mass with a hard texture and tenderness upon palpation, with no overlying erythema or induration.

Targeted ultrasound of the area revealed an irregular hypoechogenic mass measuring 0.9 × 1.7 × 1.4 cm. The origin of the mass was unclear; however, a small neck deeper to the mass was noted, indicating a possible connection with the intra-abdominal contents.

However, during the subsequent MRI three weeks after, the mass was found to have grown significantly, measuring 3.0 × 2.3 × 3.0 cm. The rapid growth of the mass during pregnancy is an important finding. The characteristics of the mass suggested a benign process, such as ectopic endometriosis or a desmoid tumor, with no evidence of local or distant extension.

## 4. Discussion

The etiology of desmoid tumors is poorly understood. The origin is often sporadic; however, an increased incidence in patients with Gardner's syndrome and familial adenomatous polyposis (FAP) has been reported [2, 3]. Desmoid tumors associated with pregnancy were first described in 1832 by MacFarlane in a postpartum woman who had an abdominal wall desmoid, which was surgically excised [4]. Due to the rarity of the condition, there are no specific guidelines on the diagnosis and management of desmoid tumors in pregnancy. The diagnosis of DF during pregnancy requires a high index of suspicion. In most cases reported, it was erroneously presumed to be uterine fibroids [5].

Multiple studies were conducted to identify the role of female sex hormones in the development of these tumors. Expression of estrogen receptors on desmoid tumors, with regression noted after menopause or following therapy with antiestrogen drugs, has been reported [6]. Various genetic, hormonal, and physical factors are considered to play an essential role in the etiopathogenesis of desmoid tumors. Mutations involving APC and CTNNB1 genes, which are part of the Wnt signaling pathway, were identified as significant genomic mutations leading to the monoclonal proliferation of fibroblasts [2, 7]. The resolution of disease with antiestrogen drugs and menopause strengthens the theory on the influence of estrogen on the development of DF [8].

Desmoid fibromatosis, although rare, tends to occur more commonly in specific contexts. These contexts include familial adenomatous polyposis (FAP), a hereditary condition characterized by the development of numerous polyps in the colon and rectum. Individuals with FAP have an increased risk of developing desmoid fibromatosis, often in the abdominal region [9]. Furthermore, Gardner syndrome, a variant of FAP, is also associated with desmoid fibromatosis [10]. Another context where desmoid fibromatosis is more prevalent is a history of trauma or previous surgery [11]. While these contexts are more frequently associated with desmoid fibromatosis, it is important to note that it can still develop in individuals without these specific risk factors, like the case in our study that develop without any syndrome or previous trauma in the region.

A review of the literature done in 2012 by Robinson et al. defined pregnancy-associated desmoid tumors as tumors discovered during pregnancy or within three years after delivery. Out of the 50 cases in the study, the most common site was the abdominal muscles, particularly the right rectus muscle [12]. There are rare cases reported of desmoid tumors in extra-abdominal sites such as the vulva, larynx, neck, and popliteal fossa [13, 14]. Furthermore, a recent study involving 382 patients with desmoid-type fibromatosis found that the prevalence of pain was only 36%, thereby highlighting the relatively uncommon nature of this symptom in the condition [15].

Although the radiologic characteristics of the mass suggested a benign process, the rapid growth resembles a malignant condition behavior, such as malignant melanoma. This is among the most rapidly growing neoplasms and one of the most commonly diagnosed during pregnancy. However, it is estimated that melanoma develops in preexisting nevi in two-thirds of cases [16]. Besides, a systematic review published in 2020 that analyzed cases of malignant melanomas during pregnancy verified that 91.6% of cases were cutaneous melanoma [17]. No skin lesions or alterations being seen on this patient associated with benign radiologic characteristics of the mass made a diagnosis of malignant melanoma less probable.

During pregnancy, a mass in the abdominal wall can also be confused with various other conditions, including uterine fibroids, abdominal hernia, and lipoma. Uterine fibroids are benign tumors that develop in the uterus and can increase in size during pregnancy, leading to noticeable protrusions in the abdominal wall [18, 19]. Abdominal hernias occur when organs or tissues protrude through abdominal wall openings, and the increased abdominal pressure during pregnancy can contribute to their development or exacerbation [20]. These hernias can resemble desmoid fibromatosis in terms of abdominal bulging. Lipomas, on the other hand, are benign tumors composed of fatty cells. Hormonal changes during pregnancy can cause lipomas to grow or become more noticeable, leading to palpable masses in the abdominal wall, which may be mistaken for desmoid fibromatosis [21, 22]. Proper differentiation between these conditions is crucial for an accurate diagnosis and appropriate treatment planning during pregnancy.

The management of desmoid tumors should be individualized. Observation with periodical imaging is the preferred method in asymptomatic patients or when the tumor is away from vital structures. Active management includes surgical excision, radiotherapy, or medical management with antiestrogen drugs such as tamoxifen or toremifene [6, 8]. The locally aggressive nature of the tumor and its unpredictable course make local control of the disease a priority, and surgical resection is recommended in most cases. The management of desmoid tumors during pregnancy is based on patient symptoms and pregnancy considerations such as gestational age when considering surgical excision. Typically, excision is postponed until after delivery [23, 24], as active surveillance is widely recognized as the primary and established strategy for managing primary or recurrent sporadic or familial desmoid tumors [3]. There have been only a few cases reported where excision was carried out during pregnancy. Durkin et al. reported a case where an extra-abdominal desmoid tumor was diagnosed during the first trimester with a core-needle biopsy of the lesion. At 20-week gestation, the patient underwent local surgical excision with clear margins and repair of the abdominal wall with mesh. The patient had an uneventful pregnancy course and delivered vaginally at term without complications [25].

Our patient noticed an abdominal lump before pregnancy. Since it had grown during pregnancy, active management was warranted to relieve the patient's pain associated with the tumor. Due to the risk of recurrence of the tumor, diligent follow-up is necessary. In a multi-institutional study done among four sarcoma centers, out of the 92 cases of DF in women, 48% had pregnancy-related DF, and 13% had a postsurgical relapse. The study concluded that even though subsequent pregnancies are associated with an increased risk of relapse, it can be safely managed [26].

In a case described by Ooi and Ngo, a patient developed a painful mass over her cesarean wound. After excision, histopathology revealed a desmoid-type fibromatosis with positive margins. An abdominal wall mesh repair was utilized. Years later, when the patient desired pregnancy, she underwent complete excision of the residual tumor before conceiving to avoid the risk of progression during pregnancy. She subsequently had an uncomplicated pregnancy and delivery [27]. This case suggests that excision of the DF tumor and abdominal wall repair is not a contraindication to subsequent pregnancy.

In our case, the patient underwent resection without mesh repair and had positive residual margins after the resection. There has been no clinical or radiological evidence of recurrence at a six-month follow-up. This study's strengths include the narrative of rare neoplasm behavior during pregnancy. In addition, unlike what is usually proposed as the recommended management, in this case, the surgical resection could not wait until delivery and needed to be done during pregnancy. Hence, besides increasing awareness of desmoid fibromatosis diagnosis, this case brings evidence that surgical management can be done safely, if necessary, during pregnancy.

Our study has limitations. Drawbacks of our study include a short follow-up period and being a single case report. The patient included may not be representative of the broader population with desmoid fibromatosis. Hence, the inclusion of only one case limits the generalizability of the findings to a larger population. Additionally, the short-term follow-up period in our study restricts our ability to assess the long-term effects of the surgical intervention and the potential recurrence of the tumor. Since the recurrence rate of desmoid fibromatosis can be as high as 80%, monitoring the patient for a more extended period could have revealed recurrence of the tumor. Furthermore, the lack of a standardized protocol for the surgical management of desmoid fibromatosis during may introduce variability in the treatment approach, making it challenging to replicate the study's results in other settings. Moreover, it is known that patients can respond differently to surgery and its inherent procedures, such as anesthesia, so it would be important to evaluate the risks and benefits on a larger scale for the development of a specific guideline for desmoid fibromatosis management during pregnancy.

A high index of suspicion is needed for the diagnosis of desmoid fibromatosis in any patient who develops an abdominal mass separate from the uterus during pregnancy. Treatment is individualized, depending on the symptoms of the patient. This case supplements the preexisting data on the effect of pregnancy on such tumors. However, surgical management of DF during pregnancy has not yet been well explored in the literature. Therefore, our case provides additional evidence that while the typical approach for managing desmoid fibromatosis during pregnancy is expectant, surgical resection should be considered based on patient's symptoms.

## 5. Patient Perspective

The patient was contacted to provide her perspective on the treatment received. The patient stated that she initially felt scared about undergoing surgical treatment during pregnancy, mainly because the specialists had advised against it. However, as the pain caused by the mass became increasingly intense and due to its rapid growth, she started to worry that the mass could affect her uterus and the growth of her child. Consequently, she made the decision to proceed with surgical resection. Her recovery from the surgery was smooth, and her pain subsided. She describes it as the best decision she could have made because it allowed her to enjoy her pregnancy without concerns or pain related to the mass.

## Data Availability

This manuscript is a case report; hence, all the information regarding the case (chart, imaging exam results, and pathology reports) contains the person's identifying data. Hence, we cannot make the information available for readers. But if necessary, we will be happy to present any data without identifying information upon request.

## Additional Points

*Key Messages*. (i) Pregnant patients who develop an abdominal mass separate from the uterus should be evaluated for desmoid fibromatosis (DF). (ii) Surgical resection of DF tumors can be safely performed during pregnancy, especially in symptomatic patients. (iii) Early diagnosis and timely surgical intervention in pregnant individuals with symptomatic DF tumors can lead to successful management and favorable outcomes for both the mother and the fetus.
